# *Ex vivo* dissection of optogenetically activated mPFC and hippocampal inputs to neurons in the basolateral amygdala: implications for fear and emotional memory

**DOI:** 10.3389/fnbeh.2014.00064

**Published:** 2014-03-05

**Authors:** Cora Hübner, Daniel Bosch, Andrea Gall, Andreas Lüthi, Ingrid Ehrlich

**Affiliations:** ^1^Hertie Institute for Clinical Brain Research and Centre for Integrative Neuroscience, University of TuebingenTuebingen, Germany; ^2^Graduate School of Neural and Behavioral Sciences, IMPRSTuebingen, Germany; ^3^Friedrich Miescher Institute for Biomedical ResearchBasel, Switzerland

**Keywords:** medial prefrontal cortex, hippocampus, amygdala, conditioned fear, optogenetics

## Abstract

Many lines of evidence suggest that a reciprocally interconnected network comprising the amygdala, ventral hippocampus (vHC), and medial prefrontal cortex (mPFC) participates in different aspects of the acquisition and extinction of conditioned fear responses and fear behavior. This could at least in part be mediated by direct connections from mPFC or vHC to amygdala to control amygdala activity and output. However, currently the interactions between mPFC and vHC afferents and their specific targets in the amygdala are still poorly understood. Here, we use an *ex-vivo* optogenetic approach to dissect synaptic properties of inputs from mPFC and vHC to defined neuronal populations in the basal amygdala (BA), the area that we identify as a major target of these projections. We find that BA principal neurons (PNs) and local BA interneurons (INs) receive monosynaptic excitatory inputs from mPFC and vHC. In addition, both these inputs also recruit GABAergic feedforward inhibition in a substantial fraction of PNs, in some neurons this also comprises a slow GABA_B_-component. Amongst the innervated PNs we identify neurons that project back to subregions of the mPFC, indicating a loop between neurons in mPFC and BA, and a pathway from vHC to mPFC via BA. Interestingly, mPFC inputs also recruit feedforward inhibition in a fraction of INs, suggesting that these inputs can activate dis-inhibitory circuits in the BA. A general feature of both mPFC and vHC inputs to local INs is that excitatory inputs display faster rise and decay kinetics than in PNs, which would enable temporally precise signaling. However, mPFC and vHC inputs to both PNs and INs differ in their presynaptic release properties, in that vHC inputs are more depressing. In summary, our data describe novel wiring, and features of synaptic connections from mPFC and vHC to amygdala that could help to interpret functions of these interconnected brain areas at the network level.

## Introduction

Emotional information is processed in distinct neural circuits. Salient emotions such as fear and anxiety are among those most intensely investigated, because resulting behaviors can be easily evoked and studied in the laboratory and the underlying brain areas are highly conserved among mammalian species from mice to man (Ledoux, 2000; Phelps and Ledoux, 2005). The most powerful models used to date to elucidate the neural circuits and mechanisms that control fear are classical Pavlovian fear conditioning and extinction of acquired fear (Maren, 2001; Ehrlich et al., 2009; Herry et al., 2010; Pape and Pare, 2010). Fear conditioning involves pairing of a previous neutral stimulus (CS) with an aversive stimulus (US), such that a CS-US association is formed. During extinction training, the CS is repeatedly presented without the US, which leads to a decrease in the learned fear response. Many lines of evidence suggest that fear and extinction learning create two distinct memory traces, and which memory is retrieved depends on the retrieval context (Bouton et al., 2006; Myers and Davis, 2007; Quirk and Mueller, 2008).

Although the amygdala is one of the most important brain areas for mediating fear and its extinction, the medial prefrontal cortex (mPFC) and hippocampus (HC), structures that are reciprocally connected to the amygdala, are implicated in aspects of acquisition, consolidation and retrieval of fear and extinction memories (Myers and Davis, 2007; Quirk and Mueller, 2008; Maren, 2011). Projections from mPFC to amygdala originate from layers 2 and 5 of different subregions, including the prelimbic (PL) and infralimbic (IL) areas and form asymmetric synapses (Pinto and Sesack, 2000, 2008). Tracing studies are not completely consistent regarding target nuclei in the amygdala. Projections from PL appear to target mainly the basal nucleus of the basolateral amygdala (BA) and portions of the capsular subdivision of the central amygdala (CeC), whereas IL projections are generally less dense and target large parts of the amygdaloid complex including the intercalated cells, and more densely a specialized lateral part of the CeC, and the ventromedial part of the LA as well as the magnocellular division of the BA (McDonald et al., 1996; Vertes, 2004; Pinard et al., 2012). Moreover, PL and IL receive amygdala projections originating mainly from the BA (Conde et al., 1995; Hoover and Vertes, 2007). Recently it has been shown that BA neurons projecting to IL and PL have opposing roles in expression of fear following extinction learning (Senn et al., 2014). Together, this raises the possibility that IL and PL may interact with the amygdala by virtue of their reciprocal connections to influence the outcome of fear and extinction learning. Projections from HC to amygdala originate in the temporal subiculum and the adjacent part of CA1. Subicular projections are dense in the accessory basal (AB) and medial part of BA, but moderate in LA and light in central amygdala, while CA1 projections to the amygdala mainly terminate in the BA, with lighter projections to LA and AB (Canteras and Swanson, 1992; Pitkanen et al., 2000). The ventral HC (vHC) is thought to contribute contextual information following extinction learning either via direct amygdala projections or indirectly by strong projections to the mPFC, which subsequently projects to the amygdala (Hoover and Vertes, 2007; Pape and Pare, 2010; Orsini et al., 2011).

Systems-level studies started to elucidate specific functions of mPFC and hippocampal regions and their interactions with the amygdala in fear learning, fear expression and extinction of fear (Maren and Quirk, 2004; Pape and Pare, 2010; Maren, 2011). For example, synchronization of activity in amygdala-hippocampal-prefrontal cortical circuits plays a critical role in anxiety, acquired fear, extinction learning, and fear discrimination (Seidenbecher et al., 2003; Lesting et al., 2011; Likhtik et al., 2014), but the underlying connectivities of neurons and microcircuits are still incompletely understood. The mPFC appears to play a double role in high and low fear states. Activation of the IL suppresses fear by suppressing amygdala output possibly via intercalated cells and central amygdala inhibition (Quirk et al., 2003; Paré et al., 2004; Maren, 2011), and/or via local BLA interneurons (Rosenkranz and Grace, 2001, 2002). The PL is thought to excite the amygdala to increase fear output during fear expression and renewal (Vidal-Gonzalez et al., 2006; Orsini et al., 2011; Sierra-Mercado et al., 2011). However, *in vivo* recordings of neuronal responses in the BLA during mPFC stimulation have yielded conflicting results about amygdala activation (Rosenkranz and Grace, 2001, 2002; Likhtik et al., 2005). Also, few data are available on how hippocampal activity influences BLA activity (Maren and Fanselow, 1995; Hobin et al., 2003; Maren and Hobin, 2007). It has been proposed that both hippocampal and PL projections to the BA mediate context-dependent fear renewal (Orsini et al., 2011), but if and how these inputs converge in the BA has not been studied.

Thus, a key open question that will guide our understanding and interpretation of systems-level functions and mechanisms, is to decipher the functional connectivities in amygdala-hippocampal-prefrontal circuits including innervation of distinct cell types, delineation of similarities or differences in synaptic input properties, and the recruitment of specific microcircuits. Here, we use an *ex vivo* optogenetic approach to study the properties of mPFC and vHC inputs to specific subtypes of BA neurons and describe distinct wiring principles and synaptic properties between these three structures.

## Materials and methods

### Animals

For all experiments, we used adult male mice (8–12 week old at time of slice recordings) of the following lines: C57BL/6J (Harlan, Netherlands), glutamate decarboxylase 67 (GAD67)–green fluorescent protein (GFP) transgenic mice (Tamamaki et al., 2003) backcrossed to C57BL/6J, and Parvalbumin-Cre (PV-Cre, Jackson stock 008069) mice crossed to Ai14 reporter mice (Jackson stock 007914) that were backcrossed to C57BL/6J. All experimental procedures were in accordance with the EU directive on use of animals in research and approved by the Regierungspraesidium Tuebingen, state of Baden-Wuerttemberg, Germany.

### Stereotactic injections

Four to six week old mice were maintained under isoflurane anesthesia, fixed in a stereotactic frame (Stoelting, USA) and injected bilaterally in either the mPFC or ventral hippocampus or a combination of both at the following coordinates from bregma (in mm). mPFC: posterior 1.9, lateral ±0.3, ventral −2.1; ventral hippocampus: posterior −3.1, lateral ±3.4, ventral −3.8. Pressure injections were performed using glass capillaries (1B150F-4, WPI, Germany) attached to a Toohey Spritzer (Toohey Company, USA). For mPFC inputs, the mPFC was injected either with 0.5 μl of rAAV-CAG-hChR2(H134R)-mCherry (serotype 2/1 or 2/9, Penn Vector Core, USA) alone or with a 0.5 μl mix of rAAV-hSyn.hChR2(H134R)-eYFP (serotype 2/9, Penn Vector Core, USA) and red retrobeads (Lumafluor, USA). For hippocampal inputs, the ventral hippocampus was injected with 0.5 μl rAAV-hSyn.hChR2(H134R)-eYFP and the mPFC was injected with 0.4 μl red retrobeads. In all cases, viral preps were diluted such that they had comparable titers (1 × 10^12^ GC/ml). Retrobeads were dialyzed against 0.32 M sucrose prior to use to avoid osmotic damage of the tissue. Four to six weeks postinjection, amygdala slices were prepared for slice recordings.

### Slice recordings

Coronal or horizontal (tilted 35° from horizontal plane) (Morozov et al., 2011) acute brain slices were prepared in ice-cold artificial cerebrospinal fluid (ACSF) supplemented with 8.7 mM MgSO_4_ at 320 μm thickness using a vibrating microtome (HM650V, Microm, Germany) equipped with a sapphire blade (Delaware Diamond Knives, USA). Slices were recovered at 37°C for 45 min and stored at room temperature in ACSF composed of (in mM): 124 NaCl, 1.25 NaH_2_PO_4_, 1.3 MgSO_4_, 2.7 KCl, 26 NaHCO3, 2 CaCl2, 18 D-glucose, 4 L-ascorbic acid and oxygenated with 95% O2, 5% CO2 until recording. Slices containing the amygdala were transferred to a submersion recording chamber, superfused with oxygenated ACSF at a speed of 1–2 ml/min, and maintained at 30–31°C. Oblique infrared and fluorescence illumination were used to target unlabeled or retrobead-labeled principal neurons, and GFP- or dtTomato-expressing interneurons for recording. Whole-cell patch-clamp recordings were performed using pipettes pulled from borosilicate glass capillaries (GB150F-8P, Science Products, Germany) with resistances of 5–8 MΩ. For most whole cell recordings, the intracellular solution contained (in mM): 130 K-Gluconate, 5 KCl, 4 Mg-ATP, 0.4 Na-GTP, 10 Na_2_-phosphocreatine, 10 HEPES, 0.6 EGTA and had an osmolarity of 290–295 mOsm and pH of 7.2–7.3. In some recordings 0.5% w/v biocytin was included in the intracellular solution. Some recordings were performed in Cs-based internal solution containing (in mM): 135 Cs-Methylsulphonate, 6 CsCl, 4 Mg-ATP, 0.4 Na-GTP, 10 Na_2_-phosphocreatine, 10 HEPES, 0.6 EGTA and had an osmolarity of 290–295 mOsm and pH of 7.2–7.3. Data were acquired using a Multiclamp 700 B amplifier, Digidata 1440 AD-board, and Clampex software (all from MDS, USA). Signals were filtered at 2 kHz and digitized at 5 kHz for synaptic current recordings and filtered at 10 kHz and digitized at 20 kHz for current-clamp recordings. Series resistance was monitored throughout each experiment and data were excluded if it changed >20%. ChR2-expressing fibers were activated with brief light pulses (0.6–2 ms, 5–10 mW/mm^2^) from a light emitting diode (470 nm, KSL70, Rapp Opto-Electronics, Germany) delivered to the whole field through the 40 × 0.8 NA objective of the upright microscope (BX51WI, Olympus, Japan). All chemicals were reagent grade (from Roth, Merck, or Sigma, Germany). CNQX was obtained from Biotrend (Germany), Picrotoxin was obtained from Sigma (Germany).

### Immunostaining and imaging

After recording, amygdala slices were fixed in 4% PFA in phosphate buffered saline (PBS) for 16–24 h at 4°C. Slices were embedded in a block of 2% Agar-Agar and resectioned at 70 μm. For visualization of projections within the amygdala, some sections were stained with Neurotrace (1:200, Invitrogen). Other sections that contained filled cells were permebealized in 0.3% Triton-X100 in PBS, and biocytin-filled cells were revealed using fluorescently-conjugated Steptavidin-Cy5 (1:200, Dianova, Germany). Immunostainings for parvalbumin were performed using standard procedures using mouse anti-Parvalbumin (Sigma, 1:2000) and Alexa-405-conjugated goat-anti-mouse (Invitrogen, 1:1000) antibody. Sections were imaged using a laser scanning confocal microscope (LSM 710, Carl Zeiss, Germany) equipped with a 25 × 0.8° NA for overview of projections or filled cells, or a 63 × 1.4° NA objective and the pinhole set to 1 airy unit for colocalization of markers in filled cells.

### Localization of injection sites and fibers

Coronal hippocampal sections were cut right after amygdala sections, immediately imaged on a fluorescent stereoscope (SCX16, Olympus, Japan) to confirm viral injection sites, and slices were fixed in 4% PFA in PBS for further analyses. The frontal cortex was removed, fixed overnight in 4% PFA in PBS, resectioned at 70 μm, and stained with Neurotrace (1:200, Invitrogen). Bead only injection sites in the mPFC were imaged using a fluorescent stereoscope. Viral and bead injection sites in the mPFC were imaged on a laser scanning confocal microscope either with a 10 × 0.3° NA or a 25 × 0.8° NA objective with the pinhole open or set to one airy unit as indicated. All images were overlaid with the mouse brain atlas (Paxinos and Franklin, 2001).

### Data analysis and statistics

All electrophysiological data were analyzed using the NeuroMatic suite of macros (http://www.neuromatic.thinkrandom.com/) and additional custom-written macros in IgorPro (Wavemetrics, USA). Input resistance (Rinput), series resistance (Rseries), membrane time constant and capacitance were calculated from 100 ms long, −5 mV voltage steps applied from a holding potential (Vhold) of −70 mV and were monitored throughout the experiment. Resting membrane potential was measured right after breaking into the cell by switching to current-clamp mode. Spiking patterns were elicited by applying depolarizing currents from 0 to +200° pA in 50° pA steps. Spike parameters were determined from the smallest current step that evoked one or a few action potentials. Spike threshold was determined as the voltage at which a >8-fold change in the rate of rise (in mV/ms) occurred. Spike amplitude was measured as the voltage difference between spike threshold and the peak of the spike. The spike half-width was measured as time difference between up- and downstroke of the spike at half-maximal amplitude. The fast afterhyperpolarization (fAHP) was measured in a 15 ms window after the peak of the spike, as the most negative membrane potential relative to the spike threshold. Synaptic current parameters were measured using Neuromatic functions on an average response generated from at least 10 individual sweeps. Amplitudes were measured as a negative or positive peak, or for late inhibitory currents as the average in a 1 ms time window 300 ms after stimulation. EPSC rise time was the time between 10–90% of maximal amplitude, and EPSC decay time was determined as time it took for the EPSC peak to decay to 37% of maximal amplitude. Latencies were measured as time between onset of stimulation and onset of the synaptic response.

Since recordings for specific group comparisons were all conducted with the same solutions and under identical conditions, we did not correct our data for the liquid junction potential. All data are reported as mean ± standard error of the mean. Statistical comparisons were performed using SPSS software (IBM, USA). For nominal data, Fisher's Exact Test or χ^2^-Test were used as indicated. Scaled data comparisons were performed using unpaired or paired Students *t*-test as indicated.

## Results

**Figure 1:**
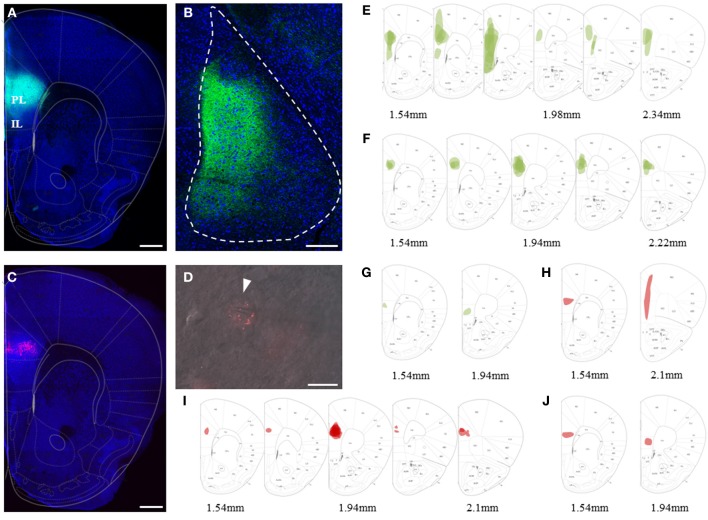
**Viral and bead injection sites for studying mPFC inputs to BLA. (A)** Confocal image of a representative brain slice of an animal injected in the mPFC with rAAV-ChR2(H134R)-eYFP (green). Scale bar: 500 μm. **(B)** Confocal image of a 35° tilted horizontal brain slice of the BLA with mPFC projections (green) corresponding to the injection site of **(A)**. Scale bar: 250 μm. **(C)** Confocal image of a representative brain slice with retrobead injection site restricted to the PL region of the mPFC (red). Scale bar: 250 μm. **(D)** Image of an *ex vivo* recorded retrogradely labeled PN in the BA. Scale bar: 10 μm. **(E–G)** Overlay of mPFC viral injection sites (green) with the mouse brain atlas for animals categorized as having main injection sites in **(E)** mPFC (*n* = 13), **(F)** PL (*n* = 17) and **(G)** IL (*n* = 2). **(H–J)** Overlay with the mouse brain atlas for animals categorized as having the main retrobead injection site in **(H)** mPFC (*n* = 2), **(I)** PL (*n* = 13) and **(J)** IL (*n* = 2).

**Figure 2:**
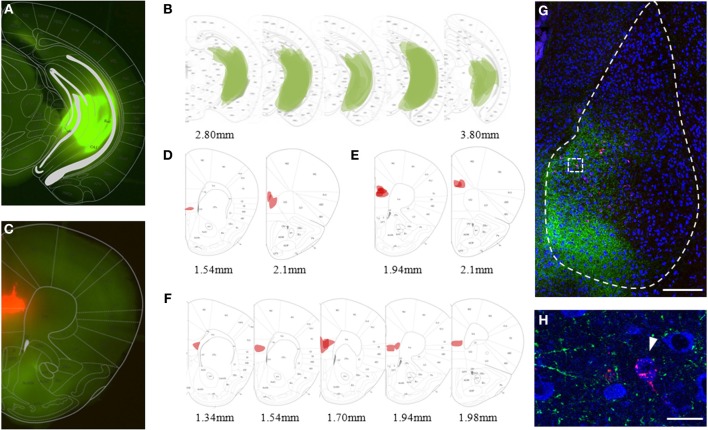
**Viral and bead injection sites for studying hippocampal inputs to BLA. (A)** Stereoscopic picture of a representative brain slice of an animal injected in the ventral hippocampus (vHC) with rAAV-ChR2(H134R)-eYFP (green). **(B)** Overlay of vHC viral injection sites with the mouse brain atlas for all animals analyzed (*n* = 23). **(C)** Stereoscopic picture of a representative brain slice with retrobead injection site in the mPFC (red) of the same animal. **(D–F)** Overlay of main retrobead injection sites with the mouse brain atlas for all animals categorized as having the main injection site in **(D)** mPFC (*n* = 2), **(E)** PL (*n* = 5) and **(F)** IL (*n* = 8). **(G)** Confocal image of a coronal brain slice of the BLA with vHC projections (green) and retrogradely labeled principle neurons projecting to the mPFC (red) of the animal shown in **(A)** and **(C)**. Scale bar: 250 μm. **(H)** Close-up of insert from **(G)** with vHC projections and retrobead-labeled neurons in the medial BA. Scale bar: 20 μm.

To dissect which neurons in the amygdala receive inputs from mPFC and vHC, to characterize properties of these inputs, and to assess activated microcircuits, we used an *ex-vivo* optogenetic approach. We injected mice with recombinant Adeno-associated virus (rAAV) expressing the light activatable protein channelrhodopsin fused to either mCherry or eYFP. Viral injections into the mPFC either infected neurons mainly located in PL or IL, or a larger area of the mPFC, encompassing PL and IL, and sometimes parts of adjacent regions (Figures 1A,E–G). For all mPFC injection conditions, dense fluorescently labeled fibers were observed in the medial BA (Figure 1B). Injections into the hippocampus were targeted toward the caudal and ventral part (vHC, Figures 2A,B) resulting in labeled fibers in the medial BA and BMA (Figures 2G,H). In some of the animals with viral injections to mPFC or vHC, we also injected a retrograde tracer (retrobeads) to label mPFC-projecting principal neurons in the BA for recording (Figures 1C,D, 2C,H). To identify interneurons in live brain slices, we used GAD-67-GFP reporter mice (Tamamaki et al., 2003), and in a few experiments PV-Cre mice crossed with a red reporter mouse (Madisen et al., 2010).

### Prefrontal and hippocampal inputs differentially recruit excitatory and inhibitory responses in BA principal neurons and interneurons

**Figure 3:**
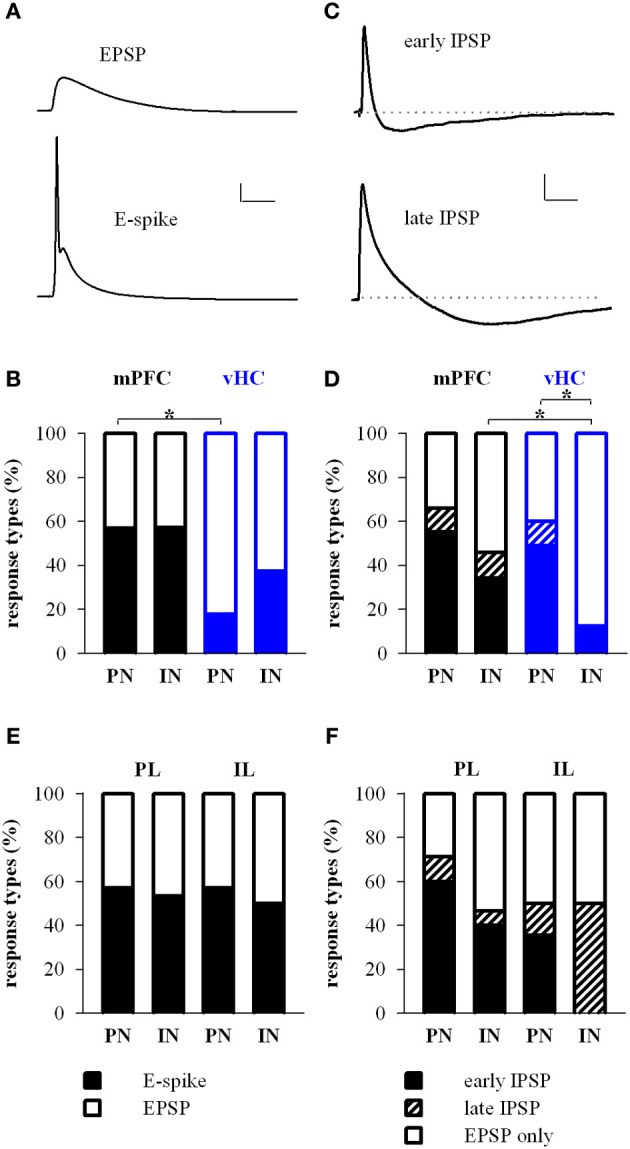
**Prefrontal and hippocampal inputs evoke excitatory and inhibitory responses in BA principal neurons and interneurons. (A)** Example traces of excitatory response types showing subthreshold EPSP and EPSP-spike (E-spike) responses. Scale bar: 10 mV/25 ms. **(B)** Relative distribution (%) of excitatory response types in principal neurons (PN) and interneurons (IN) receiving synaptic inputs from mPFC (black) and vHC (blue). Legend for **(B,E)** is shown in panel **(E)**. Spikes were more readily elicited in PNs by mPFC inputs than vHC inputs (Relative occurrence of spikes was for mPFC→PN: E-spike 57%, *n* = 65 vs. vHC→PN: E-spike 18%, *n* = 45; Fisher's Exact Test, ^*^p < 0.001). No difference was observed in INs with mPFC and vHC inputs (mPFC→IN: E-spike 57%, *n* = 35 vs. vHCI→N: E-spike 38%, *n* = 16; Fisher's Exact Test, *p* = 0.237) or INs vs. PNs within each input type (Fisher's Exact Test, *p* = 1 for mPFC and *p* = 0.164 for vHC). **(C)** Example traces showing early and late inhibitory response types. Scale bar: 2 mV/50 ms. **(D)** Relative distribution (%) of purely excitatory (open bars) and additional inhibitory response types (early IPSP: closed bars, late IPSP: striped bars) in PNs and INs receiving input from mPFC and vHC. Legend for **(D,F)** is shown in panel **(F)**. Occurrence of inhibitory response types for mPFC→PN: early 55%, late 11%, *n* = 65; mPFC→IN: early 34%, late 11%, *n* = 35; vHC→PN: early 49%, late 11%, *n* = 45; and vHC→IN: early 12%, *n* = 16. Inputs from vHC recruited IPSPs more readily in PNs compared to INs (*n* = 27/45 vs. *n* = 2/16; Fisher's Exact Test, ^*^p = 0.001). In INs, mPFC inputs recruited IPSPs more readily than vHC inputs (*n* = 16/35 vs. *n* = 2/16; Fisher's Exact Test, ^*^p = 0.028). **(E)** Relative distribution (%) of excitatory response types in neurons receiving synaptic input from prelimbic (PL) and infralimbic (IL) regions of the mPFC. Relative occurrence of spikes was for PL→PN: 57%, *n* = 35; IL→PN: 57%, *n* = 14 neurons; PL→IN: 53%, *n* = 15; IL→IN: 50%, *n* = 2. No difference in excitatory response types was observed (Fisher's Exact Test, all *p* = 1) **(F)** Relative distribution (%) of purely excitatory (open bars) and early and late inhibitory (closed and striped bars, respectively) response types in neurons receiving synaptic input from PL and IL regions of the mPFC. Occurrence of inhibitory response types for PL→PN: early 60%, late 11%, *n* = 35; and PL→IN: early 40%, late 7%, *n* = 15; IL→PN: early 36%, late 14%; *n* = 14; IL→IN: early 0%, late 50%, *n* = 2. There was no difference in inhibitory response types (Fisher's Exact Test, all *p* > 0.25).

We focused our recordings on neurons located in the medial part of the BA, the region where both, labeled mPFC and vHC axons were reliably observed in acute brain slices. Importantly, passive and active properties of principal neurons that received inputs from mPFC or the vHC were nearly indistinguishable (Table 1), and consistent with those recently described for recordings from the magnocellular region of the BA in mice (Senn et al., 2014). To study responses elicited by activation of channelrhodopsin-positive axons from the mPFC or vHC, we first recorded from principal neurons (PNs) and interneurons (INs) in current-clamp mode. As expected from a glutamatergic projection, all light-responsive PNs and INs displayed an initial depolarizing response that resembled an excitatory postsynaptic potential (EPSP, Figures 3A,B). With increasing stimulation intensity, a fraction of both PNs and INs responded with a spike arising from the EPSP (Figure 3A, bottom). In PNs, spikes were more readily elicited by mPFC- than vHC-fiber stimulation, while INs were equally likely to show spike responses for the two input pathways (Figure 3B). Under conditions where we did not elicit spikes in PNs, we observed also two types of hyperpolarizing responses, resembling fast and slow inhibitory postsynaptic potentials, which we called early and late IPSPs (Figure 3C). Stimulation of mPFC inputs elicited IPSPs in a substantial and similar fraction of PNs and INs (46–66%). In contrast, vHC afferent stimulation recruited IPSPs in a significantly larger fraction of PNs than INs (60 vs. 13%, Figure 3D). When comparing inputs, IPSPs were equally prevalent in PNs following stimulation of either mPFC or vHC inputs, whereas in INs, mPFC inputs were more likely to evoke IPSPs than vHC inputs (Figure 3D). This suggests that activation of the vHC recruits less inhibition onto INs when compared to PNs, or to neurons innervated by the mPFC.

**Table 1:** **Properties of recorded principal neurons**.

**Input type**	**mPFC**				**vHC**					**Statistical comparisons**
**Cell type**	**all PN (*n* = 65)**	**uPN (*n* = 21)**	**bPN (*n* = 26)**	**plPN (*n* = 22)**	**all PN (*n* = 45)**	**uPN (*n* = 14)**	**bPN (*n* = 24)**	**plPN (*n* = 10)**	**ilPN (*n* = 11)**	
Vrest (mV)	−69.35±0.72	−70.24±0.85	−69.38±1.22	−69.68±1.18	−67.31±0.88	−64.43±1.95^1^	−68.63±1.05^1^	−70±1.71	−67±1.58	^1^*p* < 0.05
Rinput (MOhm)	169.14±11.67	154.81±16.23	181.39±23.82	173.33±24.89	155±7.69	136.34±7.57	162.25±12.25	177.63±18.11	161.91±19.66	
Rseries (MOhm)	20.8±0.58	20.62±1.45	18.83±0.9	18.75±1.06	21.58±0.93	23.52±2.13	19.92±1.05	20.17±1.99	19.98±1.51	
Membrane tau (ms)	3.62±0.16	3.97±0.36	3.34±0.21	3.37±0.23	3.47±0.24	3.93±0.6	3.37±0.26	3.71±0.5	3.14±0.32	
Capacitance (nF)	202.6±5.77	219.30±8.34	201.12±9.54	204.48±10.37	184.01±8.24	188.58±17.37	193.25±10.3	207.76±17.52	180.16±13.72	
Excitability (Hz)	14.46±1.16	11.52±1.92	12.62±1.71	11.55±1.8	15.07±1.07	11.714±1.48	15.33±1.41	16±2.21	15.27±2.32	
Spike threshold (mV)	−38.50±0.56	−36.81±0.8	−38.81±0.92	−39.02±1.06	−38±0.6	−38.47±0.93	−37.35±0.98	−38.29±1.37	−35.84±1.69	
Spike amplitude (mV)	81.15±0.88	82.15±1.04	83.59±0.87	83.44±0.92	82.03±1.15	82.14±2.5	82.23±1.48	80.55±2.71	82.89±2.08	
Spike half-width (ms)	1.16±0.01^1^	1.15±0.03	1.16±0.02	1.16±0.02	1.23±0.03^1^	1.32±0.07	1.2±0.03	1.22±0.05	1.181±PM	^1^*p* < 0.05
fAHP (mV)	−4.77±0.3	−4.72±0.47	−4.84±0.44	−4.63±0.51	−4.49±0.36	−3.26±0.6^1^	−5.28±0.51^1^	−5.75±0.78	−5.52±0.78	^1^*p* < 0.05

*Excitability (Hz), spike frequency at 200 pA current injection; fAHP, fast afterhyperpolarization; all PN, all recorded principal neurons; uPN, unlabeled principal neuron; bPN, mPFCbackprojecting principal neuron; plPN, principal neuron projecting to prelimbic cortex; ilPN, principal neuron projecting to infralimbic cortex; values represent mean ± s.e.m. (n, number of recorded cells); statistical comparisons: superscript numbers represent pairs of significantly different values*.

In a second step, we addressed if activation of IL- vs. PL-afferents would show different response profiles, by analyzing subsets of neurons activated by fibers from localized IL and PL injections. Interestingly, we found no significant difference in excitatory or inhibitory response types onto PNs, or in the prevalence of excitatory and inhibitory input types from the PL to PNs vs. INs (Figures 3E,F). Although the dataset for IL inputs onto INs is very limited, overall our data suggest that IL and PL fiber activation leads to similar response type profiles for excitation and inhibition onto medial BA PNs and INs in naïve animals.

### Synaptic responses are comprised of early EPSCs and GABA_A_ and GABA_B_ mediated feedforward IPSCs

**Figure 4:**
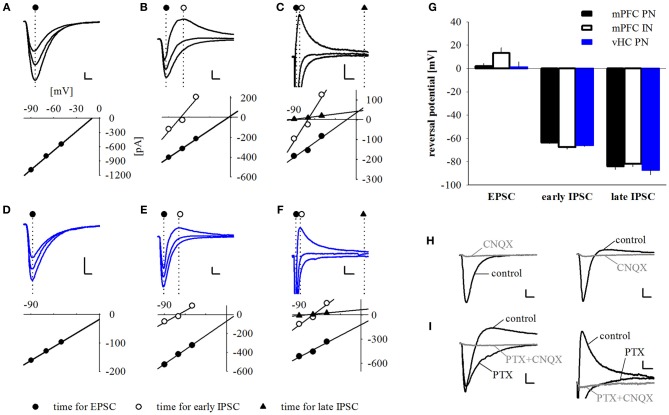
**Synaptic responses are comprised of early EPSCs and GABA_A_ and GABA_B_ mediated feed-forward IPSCs. (A–F)** Example traces and corresponding current-voltage relationship plots for pure excitatory **(A,D)**, excitatory and early inhibitory **(B,E)** and excitatory plus early and late inhibitory **(C,F)** synaptic currents elicited by either mPFC (**A–C**, black traces) or vHC (**D–F**, blue traces) afferent fiber activation. Responses were recorded at holding potentials of −90, −70 and −50 mV. Dotted lines and symbols represent time of current measurement for excitatory, early, and late inhibitory components. Scale bars for **(A,B,E)**: 100 pA/5 ms; for **(C,F)**: 50 pA/25 ms; for **(D)**: 25 pA/5 ms. **(G)** Summary graph of reversal potentials for PNs with mPFC input (EPSC: 1.88 ± 2.67 mV, *n* = 42; early IPSC: −63.65 ± 0.94 mV, *n* = 42; late IPSC: −84.06 ± 2.56 mV, *n* = 7), INs with mPFC input (EPSC: 13.33 ± 4.29 mV, *n* = 7; early IPSC: −67.44 ± 1.81 mV, *n* = 14; late IPSC: −81.73 ± 2.61 mV, *n* = 2) and PNs with vHC input (EPSC: 1.06 ± 4.74 mV, *n* = 12; early IPSC: −65.82 ± 1.09 mV, *n* = 23; late IPSC: −86.99 ± 4.12 mV, *n* = 4). **(H)** Example traces showing the effect of CNQX (10 μM) on EPSCs and IPSCs elicited by mPFC input stimulation. CNQX abolished EPSCs (left) and biphasic EPSC/IPSC responses (right). Scale bars: 50 pA/5 ms. **(I)** Example traces showing the effects of picrotoxin (PTX, 100 μM) and PTX+CNQX on EPSCs and early and late IPSCs elicited by mPFC input stimulation. PTX abolished the early IPSC and (left and right panel), but not the late IPSC (right panel). Addition of CNQX abolished the remaining EPSC (left and right panel), and late IPSC (right panel). Scale bars: 50 pA/5 ms (left) and 25 pA/25 ms (right).

**Figure 5:**
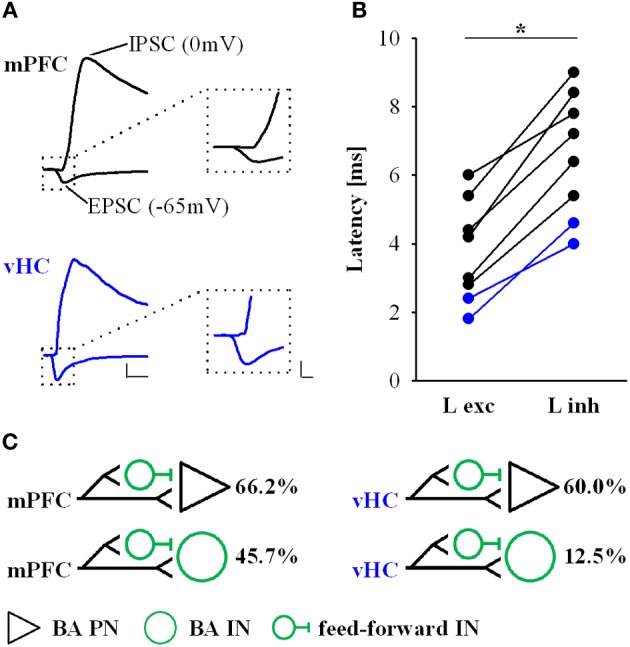
**Latencies of EPSCs and IPSCs for mPFC and vHC inputs onto BA neurons. (A)** Example traces of EPSCs and IPSCs recorded at −65 mV and 0 mV, respectively in Cs-based internal solution. Black traces represent mPFC inputs, blue traces vHC inputs onto BA PNs. Scale bar left: 200 pA/10 ms; right: 200 pA/2 ms. **(B)** Individual data points show a within-cell comparison of latencies of EPSCs (L exc) and IPSCs (L inh) in BA PNs for mPFC (*n* = 6) and vHC (*n* = 2) inputs. IPSC latencies were significantly slower that EPSC latencies (paired Students *t*-test: ^*^p < 0.01). **(C)** Proposed wiring scheme of excitatory and feedforward inhibitory connections from mPFC and vHC with neurons in the BA based on results shown in Figures 3–5. Numbers (%) represent the prevalence for recruitment of feed-forward inhibition from Figure 3D.

To confirm that light responses were generated by axonal activation and to dissect synaptic components, we performed voltage-clamp recordings. In keeping with our initial observation, we found that all PNs and INs showed light-evoked inward currents at −70 mV, which resembled excitatory postsynaptic currents (EPSCs, Figures 4A–F). These putative EPSCs were completely abolished by application of the sodium channel blocker tetrodotoxin (TTX, 1 μM), indicating that they were driven by action potentials in ChR2-expressing axons (*n* = 3, data not shown). Neurons with only EPSP-like responses in current-clamp mode displayed a single component current response with a reversal potential close to 0 mV following mPFC or vHC fiber stimulation (Figures 4A,D,G). These responses were completely blocked by the AMPA/Kainate receptor antagonist CNQX (10 μM, *n* = 3, Figure 4H left), indicating that they represent glutamatergic EPSCs. Recordings from neurons with a depolarizing and early hyperpolarizing profile revealed two current components in voltage-clamp recordings: an early component with a reversal potential close to 0 mV and a second component with a reversal potential close to −70 mV, the expected equilibrium potential for chloride, and thus GABA_A_-mediated inhibitory postsynaptic currents (IPSCs, Figures 4B,E,G). Consistent with the notion of an EPSC/earlyIPSC sequence, the second component was blocked by picrotoxin (PTX, 100 μM, *n* = 9), and the first component was blocked by subsequent application of CNQX (*n* = 6, Figure 4I, left). Furthermore, the biphasic EPSC/earlyIPSC was also completely abolished by CNQX alone (*n* = 2, Figure 4H, right), a finding that is in agreement with feedforward inhibition. Lastly, we examined neurons with a late hyperpolarization in current-clamp mode. Here, we always found three current components, the first reversing around 0 mV (consistent with an EPSC), a second reversing around −70 mV (consistent with the early IPSC described above) and a third, small component with a reversal potential close to −90 mV, the expected equilibrium potential for potassium und thus, the effector channels of GABA_B_ receptors (Figures 4C,F,G). Here, the GABA_A_ antagonist PTX only blocked the early IPSC, and subsequent application of CNQX abolished the EPSC and late IPSC (*n* = 1, Figure 4I, right). Thus, neurons with a late inhibition also display an early inhibitory response in voltage-clamp mode. We also isolated EPSCs and early IPSCs in Cs-based internal solution to precisely determine their onset latencies. Latencies of EPSC were consistently shorter than latencies of early IPSC latencies in a within-cell comparison (Figures 5A,B). Furthermore, the values are well in line with recent studies on optogenetic monosynaptic and disynaptic activation of EPSCs and IPSCs, respectively (Cho et al., 2013; Felix-Ortiz et al., 2013). Late IPSCs were not observed in Cs-based recordings, lending further support to their potassium-channel/GABA_B_ receptor based mechanism.

In conclusion, our data show that neurons in the BA receive either exclusively excitatory glutamatergic inputs or a combination of excitatory and inhibitory inputs from mPFC and vHC. Although we cannot completely exclude a contribution of feedback inhibition, our findings strongly suggest that at least early inhibition is due to feedforward processes. Feedforward inhibition was frequently observed in BA PNs for both inputs (>60%), and prominent at mPFC inputs to INs, but rarely observed for vHC inputs onto INs (Figure 5C). Postsynaptic inhibition can either be mediated by GABA_A_ receptors, or a combination of GABA_A_ and GABA_B_ receptors.

### Properties of mPFC- and vHC-evoked excitatory inputs in BA depend on input and target cell type

**Figure 6:**
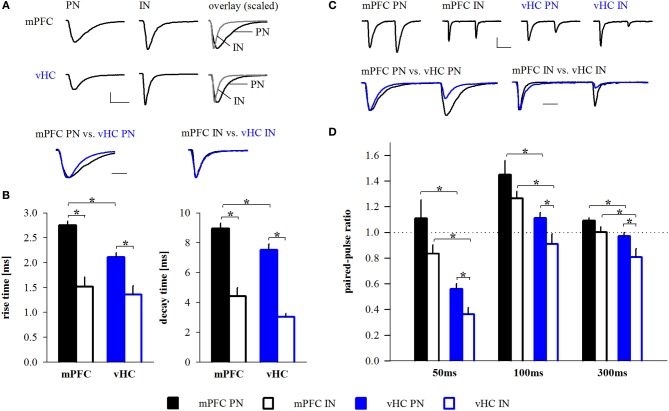
**Properties of mPFC- and vHC-evoked EPSCs in BA neurons. (A)** Example traces of EPSCs recorded at −70 mV in BA principal neurons (PN) and interneurons (IN) receiving input from mPFC (top) or vHC (middle) and amplitude-scaled overlays showing faster rise and decay of EPSCs in IN. Amplitude-scaled overlays of mPFC- or vHC-evoked EPSCs in PNs and INs show faster EPSCs of vHC inputs in PNs (bottom). Scale bars: 100 pA/10 ms (top) and 5 ms (bottom). **(B)** Summary graphs for rise and decay times of EPSCs in PNs and INs for mPFC (black) and vHC inputs (blue), all values are in Table 2 (all PNs) and Table 5 (all INs). Rise and decay times were significantly faster for mPFC inputs onto INs vs. PNs (rise time: *p* < 0.001; decay time: *p* < 0.001) and vHC inputs onto INs vs. PNs (rise time: *p* = 0.002; decay time: *p* < 0.001). vHC inputs evoked faster EPSCs in PNs than mPFC inputs (rise time: *p* < 0.001, decay time: *p* = 0.01). **(C)** Example traces of EPSCs evoked by paired pulse stimulation (interval: 50 ms) at −70 mV in PNs and INs after stimulation of mPFC (top left) or vHC (top right) inputs. Overlays scaled to the amplitude of the first EPSC illustrate differences in paired pulse ratio (bottom). Scale bars: 100 pA/25 ms (top) and 10 ms (bottom). **(D)** Summary graph for paired pulse ratios (PPR) of EPSCs at different stimulation intervals in PNs and INs for mPFC (black) and vHC inputs (blue), all values are in Table 2 (all PNs) and Table 5 (all INs). PPRs of mPFC inputs onto INs vs. PNs were not significantly different (*p* > 0.05 for all intervals). PPRs of vHC inputs onto PNs vs. INs were significantly larger (^*^p < 0.05 for all intervals). PNs and INs receiving mPFC input showed significantly larger PPRs than PNs and INs receiving vHC inputs (PN: ^*^p < 0.01; IN: ^*^p < 0.05 for all intervals).

To address if excitatory inputs onto different types of BA neurons have distinct properties, we compared EPSCs between PNs and INs in each input pathway and between input pathways. In all cases, synaptic latencies of EPSCs were consistent with monosynaptic activation (Tables 2, 5). When comparing EPSC kinetics between neuron types, INs showed more rapid rise and decay times than PNs in both input pathways (Figures 6A,B), a feature previously described for local interneurons in hippocampus and amygdala (Mahanty and Sah, 1998; Jonas et al., 2004). Consistent with that, EPSCs in INs had similarly fast kinetics when comparing mPFC and vHC inputs (Table 5, Figures 6A,B). Interestingly, when comparing PNs, we found that EPSCs evoked by vHC input had a decreased latency and slightly but significantly faster rise and decay times than those originating from mPFC inputs (Table 2, Figures 6A,B).

**Table 2:** **Properties of EPSCs onto principal neurons**.

**Input type**	**mPFC**			**vHC**			**Statistical comparisons**
**cell type**	**all PN**	**uPN**	**bPN**	**all PN**	**uPN**	**bPN**	
Amplitude (pA)	−415.7±30.03 (58)^1^	−480.88±52.33 (19)	−445.91±44.46 (24)^2^	−274.76±28.32 (42)^1^	−353.56±61.08 (12)	−250.7±33.17 (23)^2^	^1,2^*p* ≤ 0.001
Latency (ms)	2.86±0.1 (58)^1^	2.73±0.11 (19)^2^	3.13±0.19 (24)^3^	2.29±0.07 (42)^1^	2.15±0.1 (12)^2^	2.34±0.102 (23)^3^	^1,2,3^*p* ≤ 0.001
Rise time (ms)	2.75±0.09 (58)^1^	2.69±0.14 (19)^2^	2.74±0.15 (24)^3^	2.11±0.09 (42)^1^	2.0±0.09 (12)^2^	2.14±0.15 (23)^3^	^l1,2^*p* ≤ 0.001; ^3^*p* ≤ 0.01
Decay time (ms)	8.96±0.37 (58)^1^	8.37±0.38 (19)	8.59±0.6 (24)	7.52±0.39 (42)^1^	7.69±0.6 (12)	7.22±0.59 (23)	^1^*p* ≤ 0.01
PPR 50 ms	1.11±0.15 (48)^1^	0.83±0.07 (17)^2^	1.53±0.34 (19)^3^	0.56±0.05 (42)^1^	0.47±0.07 (13)^2^	0.57±0.06 (23)^3^	^1,2^*p* ≤ 0.001; ^3^*p* < 0.05
PPR 100 ms	1.45±0.11 (49)^1^	1.29±0.06 (17)^2^	1.75±0.27 (19)^3^	1.11±0.04 (42)^1^	1.02±0.07 (13)^2^	1.14±0.06 (23)^3^	^1,2^*p* ≤ 0.01; ^3^*p* < 0.05
PPR 300 ms	1.09±0.02 (46)^1^	1.11±0.04 (17)^2^	1.11±0.03 (19)^3^	0.97±0.03 (41)^1^	0.91±0.06 (13)^2^	1±0.04 (23)^3^	^1,2^*p* ≤ 0.01; ^3^*p* < 0.05
I/E ratio	0.36±0.1 (39)	0.18±0.05 (14)^1^	0.5±0.23 (16)	0.44±0.13 (25)	0.42±0.1 (7)^1^	0.45±0.18 (18)	^1^*p* ≤ 0.01

*PPR, paired-pulse ratio; I/E ratio, inhibition-to-excitation ratio; all PN, all recorded principal neurons; uPN, unlabeled principal neuron; bPN, mPFC-backprojecting principal neuron; values represent mean ± s.e.m. (n, number of recorded cells); statistical comparisons: superscript numbers represent pairs of significantly different values*.

To assess presynaptic properties, we performed paired-pulse stimulation and analysis. Because our TTX experiments suggested action potential-dependent neurotransmitter release, and ChR2(H134R) can follow stimulation frequencies up to 40 Hz reliably (Berndt et al., 2011), we used intervals between 50 and 300 ms for stimulation. At all intervals tested, we found a significant difference in the paired-pulse ratio (PPR) between mPFC and vHC inputs onto PNs and INs with consistently lower values for vHC inputs (Figures 6C,D). At the 50 ms interval, the PPR of vHC inputs was strongly depressing, suggesting a high release probability of these synapses, whereas mPFC inputs to PNs and INs showed higher values (around 1), suggesting a lower release probability (Figures 6C,D).

In our dataset, mPFC inputs to PNs evoked larger EPSCs than vHC inputs (Table 2). To rule out that the observed differences in PPR and EPSC kinetics between inputs may be due to amplitude differences, we used two approaches. Firstly, we performed amplitude-restricted analysis of EPSC properties (criterion: amplitudes <500 pA; mPFC→PN: −2.1 ± 22 pA, *n* = 40; vHC→PN: −2.0 ± 23 pA, *n* = 37; *p* = 0.081) and still detected significant differences in latency, rise, decay and PPR (*p* ≤ 0.01 for all). Secondly, we performed correlation analysis on both datasets. We found no correlation between amplitude vs. latency, kinetics, or PPR for vHC inputs (*p* ≥ 0.05 for all), and an opposite than expected correlation between amplitude and latency and amplitude and rise time for mPFC inputs (i.e., larger EPSC had faster latencies and shorter rise times, *p* < 0.05). Thus, in conclusion, differences in mPFC vs. vHC input properties did not result from amplitude differences. If faster latencies and kinetics of EPSCs in vHC inputs would result from dendritic filtering and/or synapse location, these parameters should be positively correlated. Indeed, we found a highly significant correlation between latency and rise time (*n* = 42, *R*^2^ = 0.43; *p* < 0.0001) and rise and decay time (*n* = 42, *R*^2^ = 0.21; *p* = 0.002).

In summary, EPSCs in INs displayed faster rise and decay times and lower PPRs when compared to their PN counterparts, features that would allow them to rapidly and reliably function in feedforward inhibitory circuits. Furthermore, vHC inputs are generally faster and more depressing than mPFC inputs. This is unlikely due to differences in the passive and active properties of target principal neurons (Table 1), or variability of EPSC amplitudes, but likely a feature resulting from differences in pre- and postsynaptic properties and location of specific inputs.

### Overall inhibition/excitation ratio is similar for different input types

**Figure 7:**
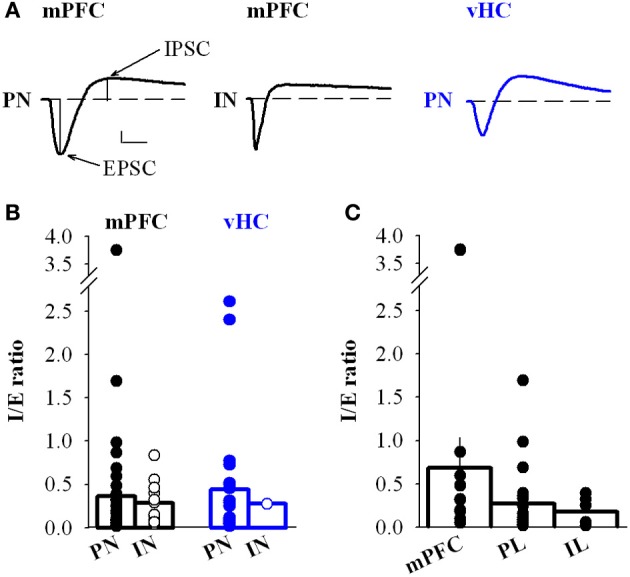
**Inhibition to excitation ratio of mPFC and vHC inputs onto BA neurons. (A)** Example traces of biphasic EPSC/IPSC sequences recorded at −50 mV in BA principal neurons (PN) and interneurons (IN) receiving input from either mPFC (black traces) or vHC (blue trace). Scale bar: 50 pA/10 ms. **(B)** Summary graph of the inhibition to excitation ratio (I/E ratio) in PNs and INs for mPFC (black) and vHC inputs (blue), all values are in Table 2 (all PNs) and Table 5 (all INs). Individual data points illustrate the high variability of I/E ratios. No significant differences were found between PNs and INs receiving mPFC input (*p* = 0.699). **(C)** Summary graph of the I/E ratio in PNs receiving inputs from subregions of the mPFC: Ratios for mPFC (undefined mPFC) 0.69 ± 0.35, *n* = 10; PL 0.27 ± 0.08, *n* = 23; and IL 0.18 ± 0.07, *n* = 6. No significant differences were found between groups (mPFC vs. PL: *p* = 0.276; mPFC vs. IL: *p* = 0.185; PL vs. IL: *p* = 0.568).

Although feedforward inhibition was observed with equal likelihood for mPFC and vHC inputs onto PNs, and also for mPFC inputs onto INs, one possibility is that the amount of inhibition could be different. We estimated the inhibitory drive by calculating the inhibition/excitation ratio (I/E ratio) from the peak amplitudes of the inward and outward components of the biphasic EPSC/IPSC recorded at −50 mV (Shin et al., 2006) (Figure 7A). The I/E ratio was highly variable, but on average not significantly different for mPFC vs. vHC inputs onto PNs (Figure 7B). There was also no difference in the I/E ratio between mPFC inputs onto PNs vs. INs (Figures 7A,B). Furthermore, activation of fibers from subregions of the mPFC (large mPFC injections vs. specific PL and IL injections) did not show any significant differences in I/E ratios (Figure 7C). Taken together, in naive animals, our data indicate no clear differences in the strength of recruited feedforward inhibition when normalized to excitation between PNs and INs, and between different input types.

### Dissection of projection neuron populations targeted by mPFC and vHC afferents

**Figure 8:**
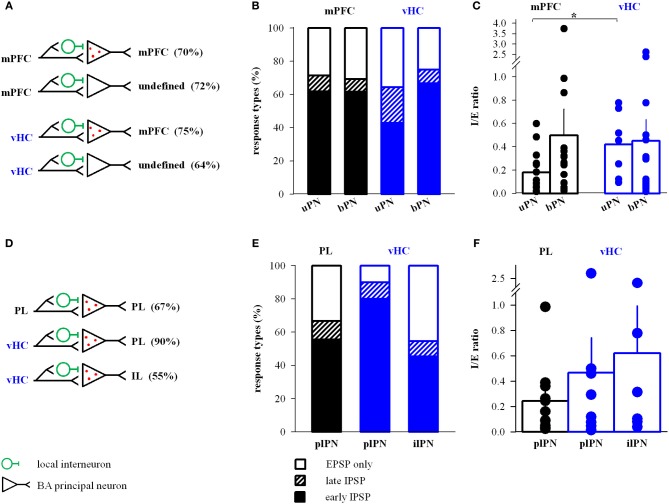
**mPFC and vHC connectivity with and properties of inputs to BA projection neurons. (A)** Scheme of projections observed onto retrobead-labeled mPFC-projecting (bPN) and unlabeled BA principal neurons (uPN). **(B)** Relative distribution (%) of purely excitatory (open bars) and additional inhibitory response types (early IPSP: closed bars, late IPSP: striped bars) in uPNs and bPNs. Occurrence of inhibitory response types for mPFC→uPN: early 62%, late 10%, *n* = 21; mPFC→bPN: early 62%, late 8%, *n* = 26; vHC→uPN: early 43%, late 21%, *n* = 14; and vHC→bPN: early 67%, late 8%, *n* = 24. No significant differences were found between inputs or cell types (Fisher's Exact Test, *p* > 0.05). **(C)** Summary graph of inhibition-to-excitation ratio (I/E ratio) in uPNs and bPNs for mPFC (black) and vHC inputs (blue). All values are in Table 2. Individual data points illustrate the high variability of I/E ratios. Synaptic inputs onto uPNs showed significantly lower I/E ratios for mPFC inputs vs. vHC inputs (uPN mPFC vs. vHC: *p* = 0.027; all other comparisons: ^*^p > 0.05). **(D)** Scheme of specific projections observed onto retrobead labeled PNs projecting to PL (plPN) and IL (ilPN) subdivisions of the mPFC. **(E)** Relative distribution (%) of purely excitatory (open bars) and additional inhibitory response types (early IPSP: closed bars, late IPSP: striped bars) in plPNs and ilPNs. Occurrence of inhibitory response types for PL→plPN: early 56%, late 11%, *n* = 12; vHC→plPN: early 80%, late 10%, *n* = 10; vHC→ilPN: early 45%, late 9%, *n* = 11. No significant differences were found between inputs or cell types (Fisher's Exact Test, *p* > 0.05). **(F)** Summary graph of I/E ratio in plPNs and ilPNs for PL (black) and vHC inputs (blue). All values are in Table 3. Individual data points illustrate the high variability of I/E ratios. No significant difference in I/E ratios was observed for mPFC or vHC inputs onto plPNs (*p* = 0.413), or vHC input onto plPNs vs. ilPNs (*p* = 0.740).

Projection neurons within the BA have diverse targets within and outside of the amygdala. In fear and extinction learning, changes in the activity of mPFC-projecting BA neurons play a critical role (Herry et al., 2008; Senn et al., 2014), while other types of projection neurons regulate anxiety-like behavior (Tye et al., 2011; Felix-Ortiz et al., 2013). Thus, we investigated properties of mPFC and vHC inputs onto mPFC-projecting BA neurons in a subset of animals that were co-injected with retrobeads in the mPFC (Figures 1, 2). We first compared mPFC-backprojecting principal neurons (bPN) with their unlabeled neighboring cells (uPN). Although we cannot rule out false-negatives among uPNs, we assume that the vast majority of these cells do not project to mPFC. Overall, both mPFC and vHC inputs to bPN and uPN displayed identical response type distributions that included excitation and feedforward inhibition (Figures 8A,B). Additionally, these distributions resembled those observed for the entire PN population for both mPFC and vHC inputs (c.f. Figure 3D, all Fisher's Exact Tests: *p* > 0.15).

When assessing properties of mPFC- and vHC-evoked EPSCs, we detected no difference between uPNs and bPNs within each input type (Table 2, all *t*-tests: *p* > 0.06). However, when comparing mPFC and vHC inputs to either bPNs or uPNs, we found the same significant changes in EPSC kinetics (except decay time), and in paired-pulse properties (Table 2). This confirms and supports our previous findings, and suggests that EPSC properties in BA PNs are determined by afferent specificity rather than projection target specificity. Interestingly, when analyzing the I/E ratio, we revealed that cells that do not project to the mPFC (uPNs) received significantly less inhibition upon mPFC compared to vHC input stimulation (Figure 8C).

We next dissected inputs and outputs of BA PNs for subregions of the mPFC. In our dataset, we found the following combinations of inputs and outputs: mPFC→PL-projecting PN (plPN) (*n* = 3), mPFC→IL-projecting PN (ilPN) (*n* = 1), IL→plPN (*n* = 2) and PL→plPN (*n* = 17), vHC→plPN (*n* = 11), and vHC→ilPN (*n* = 10). When comparing input properties in the three most frequently observed groups (PL→plPN, vHC→plPN, and vHC→ilPN, Table 3), we again revealed feedforward inhibition as a salient feature, which was particularly prevalent at vHC inputs to PL-projecting cells, but not significantly different from the overall population of PNs (c.f Figures 3D, 8D,E; Fisher's Exact Test, *p* = 0.14). Similar to the results above, properties of EPSCs appeared to be determined by input rather than output type for PNs (Table 3). Interestingly, the I/E ratio in neurons with feedforward inhibition was similar for vHC inputs onto PL- and IL- projecting cells, and similar for vHC and PL inputs onto PL-projecting cells (Figure 8F).

**Table 3:** **Properties of EPSCs onto principal neurons by specific input and output**.

**Input type**	**mPFC**			**vHC**		**Statistical comparisons**
	**PL**	**IL**	**PL**			
**Cell type**	**PN**	**PN**	**plPN**	**plPN**	**ilPN**	
Amplitude (pA)	−457.9±40.56 (33)	−316.25±64.52 (11)	−463.66±57.49 (17)^1^	−292.19±45.51 (10)^1^	−186.46±46.34 (10)	^1^*p* < 0.05
Latency (ms)	2.96±0.15 (33)	2.84±0.17 (11)	3.29±0.25 (17)^1^	2.46±0.17 (10)^1^	2.3±0.16 (10)	^1^*p* < 0.05
Rise time (ms)	2.73±0.11 (33)	3.16±0.22 (11)	2.82±0.2 (17)	2.25±0.2 (10)	2.12±0.27 (10)	
Decay time (ms)	8.71±0.44 (33)^1^	11.22±1.02 (11)^1^	8.91±0.74 (17)^2^	6.58±0.58 (10)^2^	8.01±1.17 (10)	^1,2^*p* < 0.05
PPR 50 ms	1.29±0.24 (28)	0.9±0.2 (8)	1.67±0.5 (13)	0.54±0.1 (10)	0.56±0.1 (10)	
PPR 100 ms	1.55±0.19 (28)	1.23±0.11 (9)	1.83±0.4 (13)	1.03±0.09 (10)	1.22±0.09 (10)	
PPR 300 ms	1.11±0.03 (28)	1.05±0.11 (6)	1.07±0.03 (13)	1.0±0.06 (10)	1.0±0.06 (10)	
I/E ratio	0.27±0.08 (23)	0.18±0.7 (6)	0.24±0.08 (11)	0.47±0.27 (9)	0.62±0.37 (6)	

*PPR, paired-pulse ratio; I/E ratio, inhibition-to-excitation ratio; PN, principal neuron; PL, prelimbic cortex; IL, infralimbic cortex; plPN, principal neuron projecting to prelimbic cortex; ilPN, principal neuron projecting to infralimbic cortex; values represent mean ± s.e.m. (n, number of recorded cells); comparisons: superscript numbers represent pairs of significantly different values*.

In summary, our data suggest reciprocal connections from mPFC to mPFC-projecting cells in the BA, and a pathway from vHC to mPFC via BA projection neurons that target both, PL and IL. Synaptic properties in these pathways and connections are regulated by input rather than output specificity.

### mPFC and vHC inputs target diverse classes of interneurons in the BA

**Figure 9:**
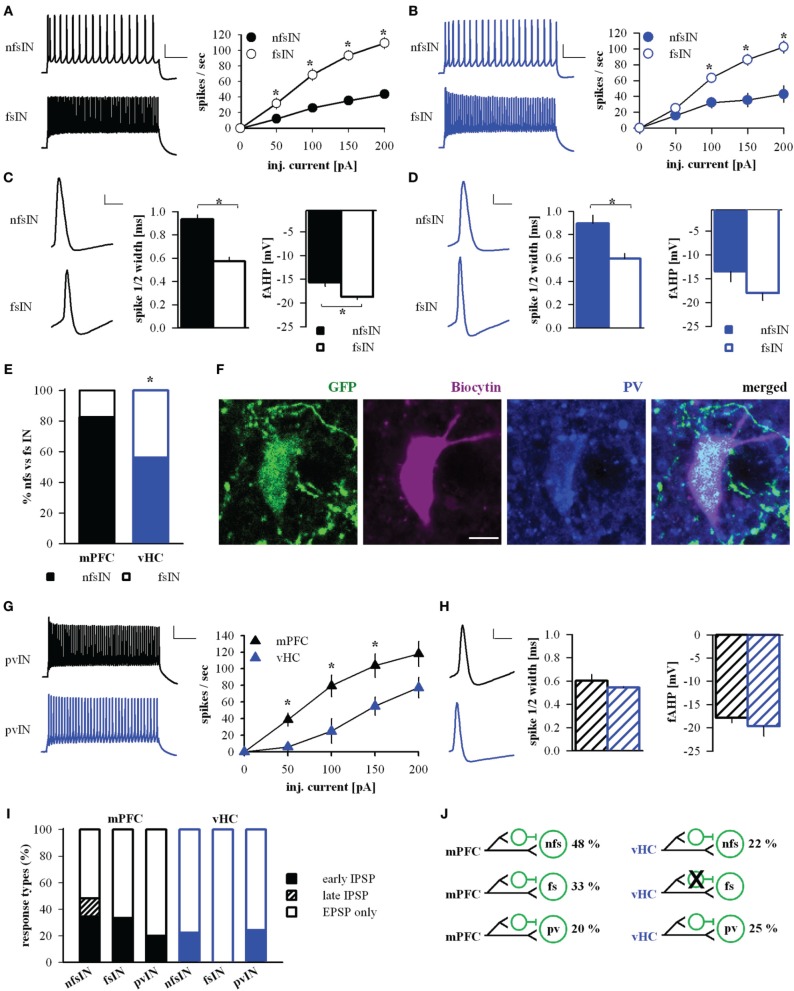
**mPFC and vHC afferents preferentially target different classes of interneurons. (A,B)** Left: Example traces of spike responses to a 200 pA/500 ms current injection in non-fast spiking (nfsINs) and fast-spiking (fsINs) INs receiving mPFC (black) or vHC input (blue). Scale bars: 20 mV/100 ms. Right: Input-output curves of INs with mPFC (black) or vHC (blue) input showed a significantly higher firing frequency in fsINs than nfsINs (^*^p < 0.05). **(C,D)** Left: Spike waveforms of nfsINs and fsINs with mPFC (black) or vHC input (blue). Scale bar: 20 mV/2 ms. Right: Graphs of spike half-width and fast after hyperpolarisation (fAHP) for nfsINs and fsINs with mPFC (black) or vHC inputs (blue), all values are in Table 4. Spike half width was broader in nfsINs vs. fsINs (mPFC: *p* < 0.001; vHC: *p* = 0.005) and the fAHP was smaller nfsINs vs. fsINs for mPFC inputs (mPFC: *p* = 0.003; vHC:*p* = 0.127). **(E)** Graph showing the distribution of BA IN-types with light responses (%). fsIN were more likely to be recruited by vHC vs. mPFC inputs (Fisher's Exact Test, *p* < 0.05). **(F)** Confocal image of fsIN recorded in a GAD67-GFP mouse (green), filled with Biocytin (pink), and identified as PV-positive (blue). Scale bar: 5 μm. **(G)** Left: Example traces of spike responses to a 200 pA/500 ms current injection in PV-positive INs from PV-Cre reporter mice (pvIN) receiving mPFC (black) or vHC inputs (blue). Scale bar: 20 mV/100 ms. Right: Input-output curves show that pvINs receiving mPFC vs. vHC inputs fire with significantly higher frequency (^*^p < 0.05). **(H)** Left: Spike waveforms in pvINs with mPFC and vHC input. Scale bar: 20 mV/2 ms. Right: Spike half-width and fAHP were similar for pvINs with mPFC or vHC input. Spike half width: 0.6 ± 0.1 ms vs. 0.5 ± 0.0 ms; fAHP −17.9 ± 1.1 vs. −19.7 ± 2.1 mV; *p* > 0.05. **(I)** Relative distribution (%) of purely excitatory (open bars) and additional inhibitory response types (early IPSP: closed bars, late IPSP: striped bars) in nfsINs, fsINs, and pvINs. Occurrence of inhibitory response types for mPFC→nfsIN: early 34%, late 14%, *n* = 29; mPFC→fsIN: early 33%, late 0%, *n* = 6; vHC→nfsIN: early 22%, late 0%, *n* = 9; and vHC→fsIN: early 0%, late 0%, *n* = 7. No significant differences were found between inputs or cell types (Fisher's Exact Test, *p* > 0.05). **(J)** Wiring scheme of different interneuron types in upon activation of mPFC and vHC afferents.

The basolateral amygdala harbors different types of local interneurons with partially distinct physiological and molecular signatures (Ehrlich et al., 2009; Spampanato et al., 2011). To address the diversity of interneurons receiving input from mPFC and vHC, we analyzed some of their passive and active properties, but found no overall differences (Table 4). However, in spike input-output curves, we observed a tendency of INs with vHC input to fire action potentials with higher frequencies (not shown). One group of INs that can be unequivocally identified electrophysiologically, are fast-spiking interneurons. Therefore, we classified individual INs as fast-spiking (fsIN) or non-fast spiking (nfsINs) based on previously published criteria including firing rate and patterns, and spike waveform (Rainnie et al., 2006; Woodruff and Sah, 2007b; Spampanato et al., 2011). Indeed, in both datasets (mPFC or vHC input) cells classified as fsINs exhibited significantly higher spike frequencies in input-output curves than nfsINs, and little spike frequency adaptation (Figures 9A,B). Furthermore, fsINs displayed a significantly shorter spike half-width and a more pronounced fast afterhyperpolarization (fAHP) (Figures 9C,D). Some of the fsINs were *post-hoc* identified as positive for the calcium binding protein parvalbumin (Figure 9F). Within the population of INs with input from mPFC, only 17% were fsINs, whereas among those with vHC input, 44% were fsINs (Figure 9E). When comparing this with the expected prevalence of fsINs of ~20% amongst all INs (McDonald and Mascagni, 2001, 2002; Woodruff and Sah, 2007b), fsIN were overrepresented in the population with vHC, but not with mPFC input (χ^2^-tests, *p* = 0.02 and *p* = 0.65, respectively). Furthermore, fsINs were more frequently targeted by vHC than mPFC afferents (Fisher's Exact Test, *p* < 0.05). When assessing inputs from specific mPFC injection regions, we observed the following prevalences in connectivity: mPFC→fsIN (*n* = 2/15, 13 %), PL→fsIN (*n* = 4/16, 25 %), IL→fsIN (*n* = 0/4, 0 %), suggesting differences between PL and IL in innervation of INs.

**Table 4:** **Properties of recorded interneurons**.

**Input type**	**mPFC**			**vHC**			**Statistical comparisons**
**Cell type**	**all IN**	**nfsIN**	**fsIN**	**all IN**	**nfsIN**	**fsIN**	
Vrest (mV)	−59.86±0.94 (35)	−59.79±1.11 (29)	−60.17±1.38 (6)	−62.31±1.3 (16)	−63.22±1.66 (9)	−61.14±2.11 (7)	
Rinput (MOhm)	506.43±40.75 (35)	522.67±46.66 (29)	427.93±73.97 (6)	429.99±46.48 (16)	495.91±66.95 (9)	345.23±50.31 (7)	
Rseries (MOhm)	33.99±1.57 (35)	34.28±1.84 (29)	32.59±2.48 (6)	31.65±1.86 (16)	34.32±2.77 (9)	28.22±1.78 (7)	
Excitability (Hz)	56.39±5.31 (31)	43.68±2.44 (25)^1^	109.33±7.67 (6)^1^	75.27±11.14 (11)	42.8±10.6 (5)^2^	102.33±7.6 (6)^2^	^1,2^*p* ≤ 0.001
Spike threshold (mV)	−35.88±0.58 (35)	−35.83±0.62 (29)	−36.12±1.76 (6)	−34.65±1.14 (16)	−34.3±1.75 (9)	−35.1±1.44 (7)	
Spike amplitude (mV)	56.18±1.46 (35)	56.38±1.7 (29)	55.23±2.37 (6)	59.45±1.94 (16)	59.88±2.4 (9)	58.89±3.39 (7)	
Spike half-width (ms)	0.87±0.04 (35)	0.93±0.04 (29)^1^	0.57±0.04 (6)^1^	0.76±0.06 (16)	0.9±0.07 (9)^2^	0.59±0.05 (7)^2^	^1^*p* ≤ 0.001; ^2^*p* ≤ 0.01
fAHP (mV)	−16.22±0.7 (35)	−15.69±0.81 (29)^1^	−18.78±0.48 (6)^1^	−15.47±1.47 (16)	−13.48±2.17 (9)	−18.03±1.5 (7)	^1^*p* ≤ 0.01

*Excitability (Hz), spike frequency at 200 pA current injection; fAHP, fast afterhypeipolarization; all IN, all recorded interneurons; nfsIN, non-fast spiking interneuron; fsIN, fast spiking interneuron; values represent mean ± s.e.m. (n, number of recorded cells); statistical comparisons: superscript numbers represent pairs of significantly different values*.

**Table 5:** **Properties of EPSCs onto interneurons**.

**Input type**	**mPFC**			**vHC**			**Statistical comparisons**
**Cell type**	**all IN**	**nfsIN**	**fsIN**	**all IN**	**nfsIN**	**fsIN**	
Amplitude (pA)	−250.45±32.05 (30)	−258.98±38.94 (24)	−216.33±39.95 (6)	−261.04±64.27 (11)	−276.14±110.91 (5)	−248.45±82.92 (6)	
Latency (ms)	2.21±0.13 (30)	2.17±0.16 (24)	2.37±0.23 (6)	2.27±0.12 (11)	2.56±0.13 (5)^1^	2.03±0.12 (6)^1^	^1^*p* < 0.05
Rise time (ms)	1.69±0.25 (22)	1.84±0.32 (17)	1.21±0.05 (5)	1.37±0.23 (9)	1.28±0.07 (4)	1.44±0.43 (5)	
Decay time (ms)	4.52±0.58 (22)	5.16±0.67 (17)^1^	2.35±0.18 (5)^1^	3.10±0.29 (9)	3.55±0.39 (4)	2.74±0.37 (5)	^1^*p* < 0.001
PPR 50 ms	0.84±0.07 (30)^1^	0.85±0.08 (24)^2^	0.79±0.11 (6)^3^	0.36±0.05 (11)^1^	0.32±0.07 (5)^2^	0.40±0.08 (6)^3^	^1^*p* < 0.001; ^2^*p* < 001;^3^*p* < 0.05
PPR 100 ms	1.26±0.06 (29)^1^	1.24±0.06 (23)^2^	1.37±0.17 (6)	0.91±0.08 (11)^1^	0.86±0.10 (5)^2^	0.96±0.12 (6)	^1,2^*p* < 0.1
PPR 300 ms	1.0±0.04 (28)^1^	0.96±0.04 (22)^2^	1.15±0.13 (6)	0.81±0.07 (11)^1^	0.72±0.08 (5)^2^	0.87±0.10 (6)	^1,2^*p* < 0.5
I/E ratio	0.29±0.07 (12)	0.30±0.07 (11)	0.13 (1)	0.28 (1)	0.28 (1)		

*PPR, paired-pulse ratio; I/E ratio, inhibition-to-excitation ratio; all IN, all recorded interneurons; nfsIN, non-fast spiking interneuron; fsIN, fast spiking interneuron; values represent mean ± s.e.m. (n, number of recorded cells); statistical comparisons: superscript numbers represent pairs of significantly different values*.

We also tested directly if mPFC and vHC inputs activate parvalbumin (PV)-expressing INs (pvINs) by recording from cells in PV-reporter mice. In a small sample, we found that mPFC afferent stimulation evoked light responses in pvINs that resembled fsINs (*n* = 5) and vHC afferent stimulation evoked light response in pvINs with more diverse firing patterns and lower average spike frequency (*n* = 4, Figure 9G). However, all targeted pvINs displayed a short spike half-width and large fAHP (Figure 9H). Light responses in pvINs had latencies of 2.5 ± 0.26 ms and 2.15 ± 0.22 ms (for mPFC and vHC input, respectively) consistent with monosynaptic activation.

We next compared response type profiles for different IN types. In agreement with findings for the overall population of INs (Figure 3D), a fraction of nfsINs, fsINs, and pvINs showed feedforward inhibition upon mPFC input activation (Figures 9H,I). In contrast, vHC afferent stimulation elicited feedforward inhibition only in ≤25% of nfsINs and pvINs (1/4 cells, not firing at high frequency), but in none of the fsINs (Figures 9I,J). In summary, mPFC and vHC inputs activate nfsINs and fsINs including PV-positive cells. Inputs from mPFC target diverse populations of INs and consistently evoke feedforward inhibition in all IN types. In contrast, vHC inputs are more likely to target fsINs, which in turn do not receive feedforward inhibitory inputs.

## Discussion

We investigated cellular and synaptic interactions between mPFC and vHC with target neurons in the medial BA, a region innervated by both areas. As expected, PNs and local INs received monosynaptic, excitatory inputs from mPFC and vHC. In addition, both inputs recruited GABAergic feedforward inhibition in a substantial fraction of PNs, but mPFC inputs more frequently recruited feedforward inhibition onto INs, suggesting activation of dis-inhibitory circuits in the BA. Amongst the innervated PNs we identify neurons that project back to subregions of the mPFC, indicating a loop between neurons in mPFC and BA, and a pathway from vHC to mPFC via BA. A general feature of both mPFC- and vHC-evoked EPSCs onto local INs is that they show faster rise and decay kinetics compared to PNs. However, mPFC and vHC inputs to both PNs and INs differ in their presynaptic properties. Our data describe wiring principles and features of synaptic connections from mPFC and vHC to amygdala that may help to interpret functional interactions of these brain areas at the network level.

### Feedforward inhibition onto BA projection neurons is a salient feature

We identified feedforward inhibition onto PNs in the BA as a prominent feature of mPFC and vHC inputs. Previous *in vivo* studies have yielded conflicting results about recruitment of local inhibition in the BLA by mPFC stimulation, possibly due to methodological constraints (Rosenkranz and Grace, 2001, 2002; Likhtik et al., 2005). Our approach is not compromised by activation of en-passant fibers or backfiring of BA projection neurons, and also allowed for detection of small inhibitory currents. We were able to recruit inhibition even at stimulation intensities that did not fire BA PNs. Together with latency and pharmacological analyses, this provides strong evidence for feedforward, rather than exclusive feedback inhibition. Thus, vHC and mPFC inputs to BA are similarly controlled by local feedforward inhibition. As at sensory inputs to the LA, this may serve to limit excitation, and to gate activity and plasticity (Li et al., 1996; Lang and Paré, 1997; Szinyei et al., 2000; Bissière et al., 2003; Shin et al., 2006).

In naïve animals, we found a highly variable inhibition/excitation ratio, even when comparing subgroups of PNs with specific inputs or outputs. This could reflect variability in inhibitory synapse location (perisomatic or proximal vs. distal dendritic), synaptic strength, number of inhibitory synapses on the target cell, or local interaction of inputs. However, we cannot rule out that this may also be partially influenced by technical variations (e.g., number of infected axons, differences in viability of INs, or IN-connectivity in slices). It is conceivable that mPFC- and vHC- driven feedforward inhibition is a substrate for plastic changes in BA inhibitory synaptic markers (Chhatwal et al., 2005; Heldt and Ressler, 2007), or in inhibitory innervation of functionally identified PNs (Trouche et al., 2013) upon fear and extinction learning.

### Identity of interneurons recruited by different inputs

Additional evidence for feedforward inhibition stems from reliable activation of local INs in the BA. In keeping with observations in other systems, EPSCs in INs displayed faster kinetics than EPSCs in PNs. This likely results from expression of glutamate receptors with fast kinetics, and enables rapid and temporally precise signaling in feedforward circuits (Jonas et al., 2004; Polepalli et al., 2010). Our data suggest that several types of interneurons are part of feedforward inhibitory circuits, including fast-spiking PV-positive (PV+) cells, and non-fast spiking PV+ and PV-negative cells. It has been proposed that fast-spiking PV+ INs are part of feedforward circuits in the BA based on their lower than expected innervation by local glutamatergic afferents (Woodruff and Sah, 2007b). On the other hand, cortical (including mPFC) innervation of PV+ cells in the BA was underrepresented compared to local innervation, suggesting that PV+ cells also participate in feedback inhibition (Smith et al., 2000). Our data may help resolve this discrepancy: We show that fast-spiking PV+ INs receive functional mPFC inputs, but fsINs are not overrepresented amongst targeted INs. In contrast, fast-spiking INs are preferentially activated by vHC afferents, but we also show that regular firing PV+ cells (Rainnie et al., 2006; Woodruff and Sah, 2007b) receive vHC inputs. This suggests that PV+ INs could be important components of vHC→BA feedforward inhibitory circuits. Overall, whether PV+ cells participate in feedforward or feedback circuits likely depends on the specific inputs that are activated. Since a majority of PV+ cells innervate BA PNs preferentially at the proximal somatodendritic domain (Muller et al., 2006; Rainnie et al., 2006), PV+ cell activation may serve to synchronize the output of PNs in the BA in response to intrinsic and extrinsic stimuli (Woodruff and Sah, 2007a).

### Differences between mPFC and vHC inputs

We discovered three major differences between inputs from mPFC and vHC: Firstly, for INs and PNs, mPFC and vHC inputs showed differences in paired-pulse responses. vHC inputs were depressing, suggesting high release probability. This, together with recruitment of feedforward inhibition could allow for low-pass temporal input filtering. In contrast, mPFC inputs had higher paired-pulse ratios, suggesting a lower release probability. Constant or facilitating excitatory inputs may be able to counterbalance recruited feedforward inhibition and to maintain sustained excitatory responses. Secondly, vHC inputs onto PNs in the BA showed shorter latencies and faster rise and decay times than mPFC inputs. The most likely explanation is that this arises from differences in dendritic input localization, because EPSC latency, rise, and decay were correlated. We cannot rule out that other parameters such as synchronization of transmitter release, or differences in glutamate receptor properties or synapse anatomy may contribute. Thirdly, we find that mPFC compared to vHC input stimulation more likely results in feedforward inhibition onto targeted INs, suggesting recruitment of dis-inhibitory circuits. We think it is unlikely that this is an artifact of our stimulation conditions, because excitatory and spike responses in INs were similarly present in both pathways, and feedforward inhibition was also similarly recruited in PNs.

### Implications for network function during fear-related behavior

It is becoming increasingly clear that the interconnected network of vHC, mPFC, and BLA subserves multiple roles in expression of fear, emotional memory, and behavioral expression of anxiety. Several distinct features of vHC inputs onto BA neurons suggest that this input is well suited to entrain synchronous amygdala activity as observed during or after fear conditioning and extinction (Seidenbecher et al., 2003; Lesting et al., 2011). For example, fast and depressing excitatory inputs in concert with feedforward inhibition put a temporal constraint on transmission of incoming activity. Additionally, activation of fast-spiking feedforward inhibitory INs may help to synchronize BA PN activity to other inputs.

The vHC has also been implicated in fear renewal and gating of fear via interactions with the PL and BA. The vHC innervates BA neurons that become active during renewal (Herry et al., 2008), and BA-projecting neurons in the vHC and PL are activated upon renewal (Orsini et al., 2011). This has lead to a model in which convergent inputs from PL and vHC drive BA activity to increase fear output. Although we have no direct evidence, the fact that PL and vHC target PL-projecting BA neurons, supports the idea of input convergence at least onto PL-projecting PNs. Our findings also delineate reciprocal connections between PL and BA PNs. In fact, a modeling study indicates that this bidirectional PL-BA loop can contribute to sustained CS-responses in PL and BA during fear expression and attributes a major role to PL inputs to BLA in this process (Pendyam et al., 2013). This fits well with our observation of non-depressing excitatory PL inputs and activation of dis-inhibitory circuits in the BA, features that could support sustained activation in PL→BA circuits. Additionally or in parallel, fear responses can also be regulated at the level of the mPFC. Here, vHC-mediated inhibition is thought to gate BLA-driven PL activation, resulting in turn in a net decrease in BLA activation and reduced fear output (Sotres-Bayon et al., 2012).

At the level of the BLA, it emerges that output specificity of BLA projection neurons also defines their behavioral role. Neurons targeting mPFC subregions or vHC have distinct roles in acquired and extinguished fear states and anxiety. For example, activity of PL- vs. IL-projecting BA cells supports the expression of high fear or low fear after extinction, respectively (Senn et al., 2014). The projection from BA to vHC controls anxiety by mediating excitation and polysynaptic inhibition in the vHC (Felix-Ortiz et al., 2013). Furthermore, vHC-projecting BA neurons also become activated during expression of acquired fear (Senn et al., 2014). Because vHC-projecting neurons are located in the magnocellular BA, the site of convergence of vHC and mPFC inputs, it is likely that their activity is also controlled by mPFC and vHC inputs to generate behavioral outputs.

We hoped to identify differences in vHC and mPFC inputs onto mPFC-projecting BA neurons (which include fear and extinction neurons) that could help to explain their responses and roles during distinct high and low fear behavioral states (Herry et al., 2008; Senn et al., 2014). However, in naïve animals, wiring of mPFC and vHC inputs and response types in PL- and IL- projecting cells and unlabeled counterparts (largely comprised of neurons projecting outside the mPFC) were similar. One important consideration is that not all PL- and IL-projecting cells are fear or extinction neurons, respectively, and input specificity could be limited to a subset of these anatomically defined neurons (Senn et al., 2014). Secondly, specific response types may only emerge through synaptic plasticity upon learning (Vouimba and Maroun, 2011; Cho et al., 2013).

Based on the opposing roles of PL and IL in fear expression and extinction one would expect differences in amygdalar activation by these inputs (Milad and Quirk, 2002; Burgos-Robles et al., 2009; Sierra-Mercado et al., 2011). Our data show that PNs in magnocellular BA are similarly activated and receive similar feedforward inhibition. This is consistent with a recent study suggesting no difference in IL and PL inputs onto BA PNs in naïve animals (Cho et al., 2013). While these authors also suggest that PL and IL inputs onto BA PNs might show equal changes upon fear extinction learning, our data imply that the impact could differ because of differential recruitment of INs that participate in feedforward circuits. Moreover, the effect of PL/IL inputs might also depend on the identity of the postsynaptic PN, and thus might have been overlooked by Cho et al. Indeed, in our hands, IL inputs target mainly non-fast spiking INs, which may be dendrite-targeting INs that either control local plasticity, or are subject to differential modulatory control (Klausberger, 2009; Spampanato et al., 2011; Chiu et al., 2013). Thus, different interneuron subtypes may participate in routing information from defined inputs to distinct and functionally diverse postsynaptic cell populations in the basolateral amygdala.

Taken together, while on the one hand our findings identify some clear differences and specializations, our data reveal a number of general and preserved features of inputs from the IL, PL, and the vHC to neurons and microcircuits in the BA. Changes in the activity of specific BA neurons upon fear and extinction learning (Herry et al., 2008; Amano et al., 2011) could then emerge either in a state-dependent manner, controlled by neuromodulators, and/or due to cellular or synaptic plasticity upon learning. This plasticity may alter recruitment of excitation, feedforward inhibition, or dis-inhibition in a cell-type specific manner. The wiring principles and synaptic features of connections from mPFC and vHC to BA described here serve as a foundation for further investigations into the roles of these inputs during fear and anxiety-related behavior, and into elucidating specific sites and mechanisms of plasticity within these circuits *in vivo* and *ex vivo*.

### Conflict of interest statement

The authors declare that the research was conducted in the absence of any commercial or financial relationships that could be construed as a potential conflict of interest.
